# Use of anticoagulants in children: a cross-sectional study at Albert Royer Children Hospital and Cardio-Pediatric Surgery Center of Fann University Hospital in Dakar, Senegal

**DOI:** 10.11604/pamj.2025.51.107.47828

**Published:** 2025-08-26

**Authors:** Awa Kane, Amadou Lamine Fall, Marie Paula Absa Dione, Hanifa Ismael Ibouroi, Indou Deme-Ly, Aminata Mbaye, Ibrahima Diop, Aida Badji, Yaye Joor Dieng, Idrissa Demba Ba, Aliou Thiongane, Papa Moctar Faye, Ibrahima Bara Diop, Ousmane Ndiaye

**Affiliations:** 1 Albert Royer Children's Hospital, Dakar, Senegal,; 2 Department of Medical, Cheikh Anta Diop University of Dakar, Dakar, Senegal,; 3 Cardio-Pediatric Surgery Center, Fann University Hospital, Dakar, Senegal

**Keywords:** Anticoagulants, children, sub-Saharan Africa

## Abstract

**Introduction:**

the use of anticoagulants in children is less frequent and requires more precautions (higher doses, unsuitable formulations, risk of treatment-related complications). We aimed to describe the challenge of using anticoagulants in children in a sub-Saharan hospital.

**Methods:**

we conducted a cross-sectional study at the Albert Royer National Children's Center and the Cardio-Pediatric Surgery Center of Fann University Hospital, with a prospective recruitment over a 6-month period. All patients aged 0 to 15 years who had received anticoagulant treatment and biological monitoring were included.

**Results:**

thirty-one patients were included. The mean age was 13 years (extremes: 13 days and 15 years) with 58.1% of girls (N=18). Among the causes of thrombotic events requiring anticoagulation, cardiac diseases were the most common in 80.6% (N=25), followed by tumor, autoimmune and traumatic causes at equal proportions of 3% (N=1). Treatment was preventive in 61.2% (N=19) of cases and curative in 38.8% (N=12). The most commonly used anticoagulant was vitamin K antagonist (VKA) in 70.9% of cases (N=22); followed by low molecular weight heparin (LMWH) in 61.29% (N=19); then unfractionated heparin (UFH) in 45.1% (N=14). One patient received rivaroxaban. All patients who received LMWH were treated with 200 IU/kg/day. The median dose of UFH was 8.3 IU/kg/h. For patients receiving VKA; 52.9% (N=9) had a dosage less than 0.1mg/kg/day. In 63.2% (N=12) of cases, the switch molecule was VKA. The median time to achieve the target INR was 13.5 days. Complications occurred in 9 patients (29.03%): heparin-induced thrombocytopenia (N=2), vitamin K antagonist overdose (N=6) and epistaxis (N=1).

**Conclusion:**

this study shows, on the one hand, that children are mostly under-dosed with anticoagulants; on the other hand, when the effective dose is reached, nearly one-third of anticoagulation-related accidents occur in this population.

## Introduction

Thromboembolic events in children are much less frequent than in adults, and occur mainly in hospitalized children, following complications of serious or chronic illnesses [1-3]. Few studies have been carried out in children on the use of anticoagulants, particularly in sub-Saharan Africa. The risk of venous thromboembolic events (VTE) in children is associated with age, with peaks in early childhood and post-puberty. Venous thromboembolic events are rare in healthy newborns, so the increased incidence in early life reflects clinical risk factors in sick newborns or premature infants, or infants with severe congenital diseases, such as heart defects [4-7]. Children frequently have several risk factors in combination, including heart disease, cancer, inflammation, trauma, surgery and medication. Immobilization is a common underlying factor in sick older children [8,9]. The prevention and treatment of thrombosis is based on several classes of anticoagulant. Heparins and related molecules have an almost immediate action, but are only available in injectable form. Anti-vitamin K agents, the most commonly used, have a delayed action and can be administered orally [9-11]. Anticoagulation in children must consider the different epidemiology of thrombotic events and changes in the coagulation system and age-dependent pharmacokinetics of anticoagulants [3].

Unfractionated heparin (UFH), low molecular weight heparin (LMWH) and vitamin K antagonist (VKA) are still the main molecules used in the preventive and curative treatment of thromboembolic events. These therapeutic classes require regular monitoring and dose adjustments to prevent the occurrence of an overdose or an accident manifested by hemorrhagic signs (epistaxis, gingivorrhagia, digestive hemorrhage, etc.) or thrombocytopenia. In addition, because of their pharmacokinetics, their use requires higher doses in children and difficult packaging [11-13]. More recently, the development of direct oral anticoagulants, which require no biological monitoring, is an interesting therapeutic option. In fact, their pharmacological properties make them particularly interesting for children: oral administration, predictable pharmacokinetics, no dependence on antithrombin, low interaction with food, and few drug interactions. However, they are still underused in our countries [14,15]. This study aimed to evaluate, in real time, the compliance of anticoagulant prescriptions in children with reference guidelines in a hospital setting in a sub-Saharan African country, and more specifically to describe the epidemiological profile of children on anticoagulants, to measure prescription compliance rates for anticoagulants in children, and to evaluate the monitoring and the occurrence of complications in children on anticoagulants.

## Methods

**Study design and setting:** this was a cross-sectional study with prospective recruitment. Between 1^st^ December 2023 and 31^st^ May 2024, during 6 months. Patients who had received anticoagulant treatment were included in the selected sites. Indications, drugs, doses, duration of treatment and monitoring have been evaluated during the study period. A data collection form records the clinical and biological data and the progress of anticoagulant treatment in children. Anticoagulant prescriptions have been compared to reference guidelines for anticoagulation in children. The study was conducted at the Albert Royer Children's Hospital, which is a national reference center for a number of pediatric conditions, including pediatric cardiology. And at the Centre Cardio-Pediatric CUOMO (CCPC) which is a national and West African sub-regional reference center dedicated to the medical and surgical management of congenital and acquired heart diseases in children.

**Study population:** children with a medical condition requiring anticoagulation were included. The inclusion criteria were: children aged 0 to 15 years who have received anticoagulation treatment and have biological monitoring of this treatment. Exclusion criteria: have no monitoring of the treatment.

**Data collection:** data were collected from medical files of hospitalized patients or outpatients, with Excel 365. Variables collected were: age, sex, clinical department, indication of anticoagulation, type of anticoagulation, dosage, changes of dosage, administration route, duration of treatments, monitoring, and evolution. Monitoring and evolution were completed in real time during the study period.

**Definitions:** the reference doses for vitamin K antagonist (VKA) were: 0,1mg/kg/d (for children > 1 year) and 0,2mg/kg/d (for children <1 year) according to the Chest 2001 guidelines for VKA. The guidelines prescriptions for UFH and LMWH were: 75 to 100 UI/kg by initial bolus, then 20 to 28 UI/kg/h for UFH and 100 to 150 UI/kg/12h for LMWH according to the Chest 2012 guidelines. Rivaroxaban dosage reference was according to the Drugs Online Database for up-to-date drug information. The target INR was between 2 and 3 for this study, according to the Chest 2001 [11,13,14,16].

**Statistical analysis:** descriptive statistics were performed with Statistical Package for the Social Sciences software (SPSS) version 26.0 and presented in percentages and frequency tables, mean, and median of the population study. Anticoagulation treatment was further analyzed by dosage groups and reported as frequencies and percentages.

**Ethical considerations:** ethical approval to conduct this study was granted by the National Ethical Committee for Health Research (reference: SEN25/47). Informed parental consent was obtained from participants.

## Results

**Figure 1:**
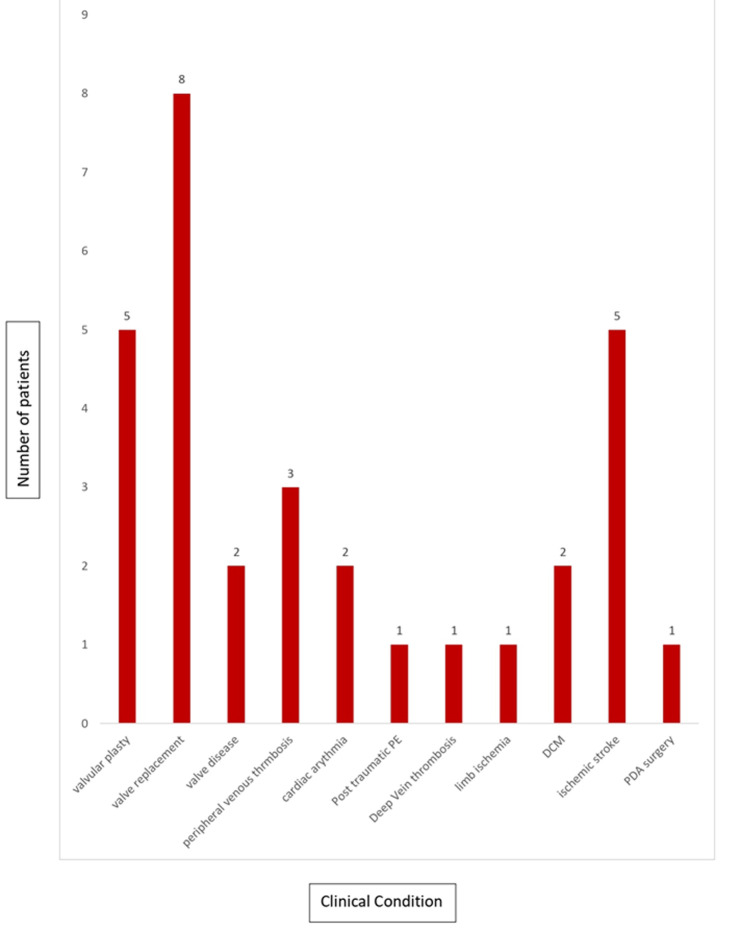
indications for anticoagulants in children

**Epidemiology of children on anticoagulant therapy:** a total of 31 patients were included. Eighteen were female (58.1%); sex ratio 0.7; with a median age of 13 years (extremes 13 days; 15 years). Five children (16.1%) were younger than 2 years of age, and the most frequent category of age was 10-15 years in 51.6% (N=16). These patients were mainly followed up at the cardiac surgery center (CCPC) in 48.3% of cases (N=15) or at the cardiopaediatric medical service of the Albert Royer Children Hospital, 25.8% (N=8). The other patients were treated in neonatology 6.4% (N=2); intensive care unit in 6.4% (N=2); dermatology in 6.4% (N=2); pediatric surgery in 3.2%; (N=1) and pneumology 3.2% (N=1) departments. Anticoagulants were used in this study for the treatment of arterial or venous thrombotic events or for preventive purposes. Among the causes of thrombotic events requiring anticoagulant treatment, cardiac causes were the most common, accounting for 80.6% of cases (N=25), followed by tumor, autoimmune and traumatic causes, equally divided at 3.2% (N=1). Cardiac causes were represented by heart valve disease in 68% (N=17), tetralogy of Fallot in 12% (N=3), dilated cardiomyopathy in 16% (N=4) and persistent ductus arteriosus in 4% (N=1). The main thromboembolic events that occurred in patients with heart disease are listed in Figure 1.

**Anticoagulant treatment: drugs, dosage, administration route, monitoring:** four (4) anticoagulants were used during the study. The first-line treatment was vitamin K antagonists (VKA), acenocoumarin in 12.9% of cases (N=4); enoxaparin (LMWH) in 22.58% of cases (N=7); unfractionated heparin in 45.2% (N=14), LMWH associated with VKA in 16.10% of cases (N=5) and a direct oral anticoagulant (DOAC), rivaroxaban 3.2% (N=1). This treatment was preventive in 61.2% of cases (N=19) and curative in 38.8% of cases (N=12). Switching was carried out in 19 patients, i.e. 61.2% of cases. In 63.2% (N=12) of cases the switch molecule was acenocoumarin (VKA) and in 36.8% (N=7) the switch molecule was LMWH. Twelve patients had not been switched: 7 patients (58.3%) on UFH for valve replacement. Two patients (16.6%) were initially on VKA, 2 others on LMWH and one patient on AOD for peripheral venous thrombosis due to cancer. Table 1 shows the dosage in mg/kg/d used by caregivers for VKA (acenocoumarin) Table 1. All patients who received LMWH were given a dose of 200 IU/kg/day. The median dose of UFH was 200 IU/kg/day, i.e. approximately 8.3 IU/kg/h. The only patient receiving Rivaroxaban was on a dose of 30 mg/day. He was undergoing surgery, was 7 years old and weighed 23 kg. Patients on LMWH received this treatment subcutaneously. Unfractionated heparin was administered intravenously using an electric syringe pump. Rivaroxaban and VKA were administered orally. The VKA was repackaged in powder form by a hospital pharmacy and distributed in sachets for children too young to swallow tablets. The duration of treatment for patients who had undergone short-term anticoagulation for preventive purposes was for patients who had undergone valve replacement surgery. One patient was on anticoagulation for 6 months for a pulmonary embolism. Patients taking anticoagulants for between 3 and 6 months were being treated for venous or arterial thrombosis. Table 2 shows the duration of anticoagulation of participants Table 2. The median time between starting treatment and achieving the target International Normalized Ratio (INR) was 13.5 days, with extremes of 3 days and 120 days. The target INR was between 2 and 3 for all patients. The median number of INR determinations before the therapeutic target was reached was 3, with extremes of 6 and 1. The INR was measured once in patients taking rivaroxaban. Table 3 shows the distribution of patients according to INR results obtained during the study. No patients were tested for anti-Xa activity during the study period.

**Table 1:** dosage of VKA in mg/kg

Acenocoumarin posology in children in mg/kg	Number	Percentages (%)
< 0.1 mg/kg	9	52.9
Between 0.1 et 0.2 mg/kg	7	41.2
At 0.2 mg/kg	1	5.9
Total	17	100

VKA: Vitamin K antagonist

**Table 2:** duration of anticoagulation of the study’s participants

Duration of anticoagulant treatment during the study period	Number	Percentages (%)
Between 3 to 8 days	8	25.8
< 3 months	2	6.5
Between 3 to 6 months	8	25.8
6 months	1	3.2
Continuous (life long)	12	38.7
Total	31	100

**Table 3:** distribution of patients according to INR results obtained during the study

Dosage INR	INR 1	INR 2	INR 3	INR 4	INR 5
< 2	26	16	5	6	3
Between 2 and 3	3	7		3	6
Between 3 and 4	1	2	3	0	1
Overdose	0	1	2	3	0
Total	30	26	15	12	10

INR: International normalized ratio

**Progression with treatment:** during the study, 3 types of complications related to anticoagulation were recorded. These complications occurred in 9 patients, i.e. 29.03% of cases. These were heparin-induced thrombocytopenia in 2 patients treated for ischemic stroke in cardiac disease, such as tetralogy of Fallot. The third was a patient with rheumatic heart disease who had been started on UFH. Six patients presented with VKA overdose, and in all of these patients anticoagulation was temporarily stopped and vitamin K was systematically administered by injection. Only one patient suffered an epistaxis during the study, taking VKA for a stroke following tetralogy of Fallot. He had an INR of 3.96 at the time of this event. The outcome was favorable in 93.5% of cases (N=29). Two deaths were recorded: a neonate with peripheral venous thrombosis of no known cause who had received LMWH, and an adolescent girl who died of organic renal failure requiring an intraoperative dialysis session for a valve prosthesis who was on UFH. There were no hemorrhagic events reported in patients taking rivaroxaban during the 2 months of treatment. During the study period, there was no subsequent monitoring after the target INR was reached, except for one patient.

## Discussion

The aim of this study was to describe the use of anticoagulation in children at the Albert Royer Children´s Hospital and at the Centre Cardio-Pediatric CUOMO (CCPC). Results of this study showed that children on anticoagulants were mostly between 10 and 15 years of age (51.6%; N=16). These patients were mostly under-dosed with AVK (52.9%; N=9). However, when the effective dose was reached, nearly one-third of anticoagulation-related accidents occurred in this population (29.03%; N=9). Two peaks in incidence have been described in children: the neonatal period and adolescence [3,17,18]. In our study, the second peak was in adolescence, with the age group 10 to 15 years being the most represented and the median age of patients being 13 years. However, we did not observe a peak before one year of age, although two newborns were included in the study. This could be explained by the lesser use of central catheters in neonates in limited-resources settings, which could lead to under-diagnosis in this age group. In our study, congenital or acquired heart disease accounted for 83% of the risk factors for thrombotic events requiring anticoagulation. In a review by Chalmers in 2006, cardiac causes (8 to 19%) ranked 3rd to 5th after venous catheters (33 to 48%), sepsis (7.3 to 46%), cancer (8 to 26%) and surgery (5.8 to 15%). This different distribution from that found in our study may be explained by the study sites being centers of reference in pediatric cardiology, which could explain this over-representation of cardiac causes, although other causes such as tumor, trauma and autoimmune disease were also found [6,19].

Unfractionated heparin is mainly used in patients requiring rapid or patients with central catheters. The major risk associated with its use is the occurrence of bleeding, which varies between 1.5 and 24% of cases. In our study, UFH was used in 22.5% of cases of cardiac surgery. This is consistent with the indications set out by Radulescu in 2017 [20]. We reported one case of HIT in a patient who had received UFH, i.e. 3.3% of cases; this is broadly comparable to prevalence in children from 0.05 to 3.7% [21-24]. We did not note any post-injection bleeding with UFH, although the dosage used in the study was lower than recommended doses [11,25]. None of the patients had an assay of anti-Xa activity, which is the recommended test for monitoring the efficacy of treatment [26,27]. Low molecular weight heparin was the second most commonly used first-line treatment, either alone or in combination with VKA (41.93% of cases); the dosage and method of administration used in the study complied with international recommendations, except in neonates where the dose used was lower, 100 IU/Kg/12H vs 150 IU/kg/12H [13]. This could be explained by the exhaustive precautions taken by practitioners for this age group and by the fact that they did not want to incur a risk of bleeding in these already seriously ill neonates. In our study, we recorded 2 cases of HIT in patients on LMWH. Although the risk of HIT has been described in patients on LMWH, it is lower than in those on UFH. In our study, only 1 patient on UFH developed HIT, which may be explained by the fact that patients on UFH were under-dosed, whereas those on LMWH were at the correct therapeutic dose, with the exception of neonates.

The vitamin K antagonist was the drug most frequently administered to patients during the study (70.9% of cases). Vitamin K antagonist doses at the start of treatment were lower than the Chest 2001 recommendations, i.e. 0.2mg/kg/day in 94.1% of cases (N=16) [13]. This could be explained by the habit of practitioners to use the lower adult doses without considering the age-dependent pharmacokinetics of VKAs. This practice results in a longer time to reach the target INR, with a median time of 13 days in our study, in contrast to a timeline of 7 days in Morocco [28]. The average number of INR tests before the target INR was reached was 3, and only one patient was rechecked after the target INR was reached. This can be explained by the cost of this test, which was not reimbursed. We recorded one case of bleeding in the form of epistaxis in a patient taking VKAs. We recorded 2 cases of death during the study, i.e. 6.4% of cases, including one unrelated to anticoagulation, i.e. 3.2% of direct mortality. Our results are comparable to those found in Canadian, and British registries, which give a rate of 9 to 17% for overall mortality and 1.5 to 2.2% for mortality directly attributable to thromboembolic events [6,17,19]. In our study, only one patient undergoing surgery was started on rivaroxaban at a dose of 30 mg/d. Although this dosage was high, no minor or major bleeding was reported in this patient during the course of treatment (2 months) [3,14]. This demonstrates the advantages and safety of these new anticoagulants, which are still not widely used in our countries. Rivaroxaban has the advantage of not requiring biological monitoring, which is a cost for our patients. In addition, unlike VKAs, rivaroxaban is available as a suspension and presents a lower risk of major bleeding than VKAs [29,30]. The use of anticoagulants in children has been little studied in sub-Saharan Africa, and this study shows that more than half of children are often under-dosed, and that almost a third are exposed to anticoagulation-related complications after reaching therapeutic doses. However, these observations are limited by the small size of our study sample; hence, a need for further studies with more participants.

## Conclusion

Anticoagulants are still molecules with several indications in pediatrics. Cardiac causes are among the most frequent. This study shows that it takes longer to reach therapeutic targets with anticoagulants and that pediatrician often under-dose anticoagulants, without taking into account age-dependent pharmacokinetic parameters in children. Complications related to the use of anticoagulation in children occur in nearly a third of cases after reaching the therapeutic dosage; however, mortality directly related to anticoagulation remains very low. This study also highlights the underutilization of a new class of anticoagulant: direct oral anticoagulants, which do not require biological monitoring and are therefore less costly for the patient. The availability and access of these direct oral anticoagulants would be an interesting therapeutic option in the context of countries with limited resources.

### What is known about this topic

- Children require higher doses of anticoagulants compared to adults according to guidelines;
- All the classical anticoagulants require regular monitoring to prevent bleeding or relapse;
- Safety and efficacy of direct oral anticoagulants have been demonstrated to be comparable to standard of care anticoagulants without the need for monitoring.

### What this study adds

- Classical anticoagulants are used at dosages lower than recommended, leading to a delay in reaching therapeutic targets and repeated monitoring;
- Although anticoagulant accidents occur in about a third of cases, mortality directly related to the use of anticoagulation remains low even in sub-Saharan setting;
- Direct oral anticoagulant is under-used in children in countries with limited resources, although it is an interesting therapeutic alternative.
